# Gain of chromosome arm 17q is associated with unfavourable prognosis in neuroblastoma, but does not involve mutations in the somatostatin receptor 2 (SSTR2) gene at 17q24

**DOI:** 10.1038/sj.bjc.6692231

**Published:** 1999-12

**Authors:** F Abel, K Ejeskär, P Kogner, T Martinsson

**Affiliations:** Department of Clinical Genetics, Sahlgrenska University Hospital/East, Gothenburg S-416 85, Sweden; Childhood Cancer Research Unit, Karolinska Institute, Karolinska Hospital, Stockholm S-171 76, Sweden

**Keywords:** 17q, SSTR2, neuroblastoma

## Abstract

Deletion of chromosome arm 1p and amplification of the MYCN oncogene are well-recognized genetic alterations in neuroblastoma cells. Recently, another alteration has been reported; gain of the distal part of chromosome arm 17q. In this study 48 neuroblastoma tumours were successfully analysed for 17q status in relation to known genetic alterations. Chromosome 17 status was detected by fluorescence in situ hybridization (FISH). Thirty-one of the 48 neuroblastomas (65%) showed 17q gain, and this was significantly associated with poor prognosis. As previously reported, 17q gain was significantly associated with metastatic stage 4 neuroblastoma and more frequently detected than both deletion of chromosome arm 1p and MYCN amplification in tumours of all stages. 17q gain also showed a strong correlation to survival probability (*P* = 0.0009). However, the most significant correlation between 17q gain and survival probability was observed in children with low-stage tumours (stage 1, 2, 3 and 4S), with a survival probability of 100% at 5 years from diagnosis for children with tumours showing no 17q gain compared to 52.5% for those showing 17q gain (*P* = 0.0021). This suggests that 17q gain as a prognostic factor plays a more crucial role in low-stage tumours. Expression of the somatostatin receptor 2 (SSTR2), localized in chromosome region 17q24, has in previous studies been shown to be positively related to survival in neuroblastoma. A point mutation in the SSTR2 gene has earlier been reported in a human small-cell lung cancer. In this study, mutation screening of the SSTR2 gene in 43 neuroblastoma tumours was carried out with polymerase chain reaction-based single-stranded conformation polymorphism/heteroduplex (SSCP/HD) and DNA sequencing, and none of the tumours showed any aberrations in the SSTR2 gene. These data suggest that mutations in the SSTR2 gene are uncommon in neuroblastoma tumours and do not correlate with either the 17q gain often seen or the reason some tumours do not express SSTR2 receptors. Overall, this study indicates that gain of chromosome arm 17q is the most frequently occurring genetic alteration, and that it is associated with established prognostic factors. © 1999 Cancer Research Campaign


*British Journal of Cancer* (1999) **81**(8), 1402–1409
© 1999 Cancer Research Campaign
Article no. bjoc.1999.0859

### Gain of chromosome arm 17q is associated with unfavourable prognosis in neuroblastoma, but does not involve mutations in the somatostatin receptor 2 (SSTR2) gene at 17q24

**F Abel[1], K Ejeskär[1], P Kogner[2] and T Martinsson[1]**

[1]Department of Clinical Genetics, Sahlgrenska University Hospital/East, S-416 85 Gothenburg, Sweden; [2]Childhood Cancer Research Unit, Karolinska Institute, Karolinska Hospital, S-171 76 Stockholm, Sweden

**Summary** Deletion of chromosome arm 1p and amplification of the MYCN oncogene are well-recognized genetic alterations in neuroblastoma cells. Recently, another alteration has been reported; gain of the distal part of chromosome arm 17q. In this study 48 neuroblastoma tumours were successfully analysed for 17q status in relation to known genetic alterations. Chromosome 17 status was detected by fluorescence in situ hybridization (FISH). Thirty-one of the 48 neuroblastomas (65%) showed 17q gain, and this was significantly associated with poor prognosis. As previously reported, 17q gain was significantly associated with metastatic stage 4 neuroblastoma and more frequently detected than both deletion of chromosome arm 1p and MYCN amplification in tumours of all stages. 17q gain also showed a strong correlation to survival probability ($P = 0.0009$). However, the most significant correlation between 17q gain and survival probability was observed in children with low-stage tumours (stage 1, 2, 3 and 4S), with a survival probability of 100% at 5 years from diagnosis for children with tumours showing no 17q gain compared to 52.5% for those showing 17q gain ($P = 0.0021$). This suggests that 17q gain as a prognostic factor plays a more crucial role in low-stage tumours. Expression of the somatostatin receptor 2 (SSTR2), localized in chromosome region 17q24, has in previous studies been shown to be positively related to survival in neuroblastoma. A point mutation in the SSTR2 gene has earlier been reported in a human small-cell lung cancer. In this study, mutation screening of the SSTR2 gene in 43 neuroblastoma tumours was carried out with polymerase chain reaction-based single-stranded conformation polymorphism/heteroduplex (SSCP/HD) and DNA sequencing, and none of the tumours showed any aberrations in the SSTR2 gene. These data suggest that mutations in the SSTR2 gene are uncommon in neuroblastoma tumours and do not correlate with either the 17q gain often seen or the reason some tumours do not express SSTR2 receptors. Overall, this study indicates that gain of chromosome arm 17q is the most frequently occurring genetic alteration, and that it is associated with established prognostic factors. © 1999 Cancer Research Campaign

**Keywords**: 17q; SSTR2; neuroblastoma

Neuroblastoma, a malignant tumour of the sympathetic nervous system, is the most common extracranial solid tumour of childhood (Gale et al, 1982). The clinical behaviour of neuroblastoma varies from spontaneous regression to malignant progression. It can also differentiate into a benign ganglioneuroma in some older patients. Generally, favourable outcome is associated with young age of diagnosis, but biological differences of the tumours in the age groups appear to be very important as well (reviewed in Brodeur, 1998).

A number of genetic alterations has been shown to be associated with poor prognosis, and these have been used as genetic markers of neuroblastoma. The two most important types of somatic abnormalities are deletion of the distal part of the short arm of chromosome 1 (1p), and amplification of the MYCN oncogene. Both MYCN amplification and 1p deletion are associated with rapid progression and poor prognosis (Seeger et al, 1985; Ambros et al, 1995; Caron et al, 1996). DNA content of neuroblastoma has been shown to be associated with prognosis as well. Tumours with increased DNA content (or 'polyploid' karyotype) is associated with a favourable outcome in infants with neuroblastoma (Look et al, 1991). Recently, focus has been directed towards a third karyotypic abnormality in neuroblastoma tumours, i.e. extra copies of the distal part of the long arm of 17 (17q gain). 17q gain is defined as a structural rearrangement of chromosome 17, which results in partial gain of 17q relative to 17p. This almost invariably involves a breakpoint in 17q12–21 with gain of the distal portion of the 17q arm (Lastowska et al, 1997*a*). Unbalanced translocation resulting in loss of distal 1p and gain of 17q has previously been identified by fluorescence in situ hybridization (FISH) in primary tumours and neuroblastoma cell lines (Caron et al, 1994; Van Roy et al, 1994). It was also demonstrated by Southern blot and cytogenetic analysis that the translocation very likely takes place in the S/G2 phase (Caron et al, 1994). More recently, comparative genomic hybridization (CGH) studies have shown that extra chromosome 17q material occurs in approximately 70–80% of primary tumours, either as gain of the whole chromosome 17 or the q arm alone (Plantaz et al, 1997; Lastowska et al, 1998; Vandsompele et al, 1998). Using FISH studies Meddeb et al (1996) and Lastowska

*Received 9 February 1999*
*Revised 27 May 1999*
*Accepted 8 June 1999*

*Correspondence to:* T Martinsson et al (1997*b*) showed that 17q can translocate on to a number of chromosome regions other than 1p, and that such rearrangements also results in gain of 17q material. Meddeb et al (1996) reported that among translocations only 17% were (1;17) translocations, whereas the remainder involved various other chromosomes: chromosome 4, 5, 6, 9, 11, 12, 14, 17 and 19. Moreover, both groups found that 17q gain also showed correlation to poor prognosis. A third study showed that 17q gain is associated with all established indicators of poor prognosis; stage 4 disease, age above 1 year at diagnosis, 1p deletion, MYCN amplification and diploidy/tetraploidy (Lastowska et al, 1997*a*). Lastowska et al (1997*a*) and Caron et al (1996) also found that 17q gain was associated with poor outcome.

Somatostatin (SST; synonym name 'somatotropin release inhibitory factor', SRIF), a growth hormone inhibitory substance, is a multifunctional peptide that is distributed widely throughout the central nervous system (CNS) and peripheral tissues (reviewed in Bell et al, 1995; Patel et al, 1995). At cellular level, actions of SST is involved in many different processes, including cell proliferation (Patel et al, 1995). Recently, it was shown by radioimmunoassay that somatostatin in neuroblastoma is associated with differentiation to benign ganglioneuroma in vivo and favourable outcome in advanced tumours (Kogner et al, 1997). The physiological actions of somatostatin are mediated by high-affinity receptors on the surface of responsive cells that are coupled by G proteins to a multiple effector system including adenylyl cyclase, ion channels and tyrosine phosphatases. Five somatostatin receptors subtypes, SSTR 1–5, have been cloned (Yamada et al, 1992). The somatostatin receptor isoform 2 gene (SSTR2) is mapped to 17q24 and encodes a 369 amino acid membrane protein (Yamada et al, 1992). Recently, in vivo expression of SSTR2 in neuroblastoma tumours was shown to be correlated with young age at diagnosis, localized clinical stage and favourable outcome (Schilling et al, 1999). In another study, Sestini et al (1996) found that SSTR2 expression in vitro was negatively related to MYCN amplification and according to Kaplan–Meier analysis positively related to survival. Interestingly, Zhang and associates found a point mutation in the SSTR2 gene in human small-cell lung cancer cell line COR-L103 (Zhang et al, 1995). The mutation caused loss of the C-terminal amino acid residue number 182 of SSTR2.

The aims of this study were to investigate the occurrence of 17q gain in the patient material of the Swedish neuroblastoma tumour study group, and its relation to survival and poor prognostic factors such as MYCN amplification, 1p deletion and diploid DNA content. Moreover, the aims were to investigate the role of the SSTR2 gene in neuroblastoma development and progression, by screening of DNA from neuroblastoma tumours for possible mutations in the SSTR2 gene.

**Table 1.** Summary of clinical parameters and experimental data from patients with neuroblastoma compared to 17q gain

| Patients | Stage | Sex | 1p-del | N-myc | 17q gain | Outcome | Survival |
|---|---|---|---|---|---|---|---|
| 84 | 1 | F | – | – | – | N | 97+ |
| 124 | 1 | M | nd | – | – | N | 61+ |
| 146 | 1 | F | – | – | – | N | 80+ |
| 156 | 1 | M | – | – | – | N | 75+ |
| 161 | 1 | F | – | – | – | N | 68+ |
| 181 | 1 | F | – | – | + | N | 47+ |
| 51 | 2A | F | – | – | – | N | 134+ |
| 121 | 2A | M | + | – | + | D | 96 |
| 177 | 2A | M | – | – | – | N | 13+ |
| St156 | 2A | F | – | – | + | N | 2+ |
| St124 | 2 | M | + | – | + | A | 26+ |
| 127 | 2B | F | – | – | + | N | 87+ |
| 138 | 2B | M | – | – | + | N | 79+ |
| 125 | 4S | M | nd | – | – | N | 89+ |
| St142 | 4S | M | (+) | + | + | D | 0 |
| 69 | 3 | M | – | – | + | D | 17 |
| 85 | 3 | F | – | – | – | N | 98+ |
| 128 | 3 | M | nd | nd | – | N | 89+ |
| 135 | 3 | M | nd | + | + | D | 6 |
| 136 | 3 | F | nd | + | + | D | 11 |
| 153 | 3 | F | – | – | – | N | 74+ |
| 157 | 3 | M | – | – | – | DOC | 0 |
| 187 | 3 | F | – | – | + | A | 7+ |
| St99 | 3 | F | + | – | + | D | 10 |
| St100 | 3 | F | – | – | – | N | 47+ |
| St131 | 3 | M | + | + | + | L | L |
| St164 | 3 | M | – | + | + | A | 7+ |
| 32 | 4 | M | – | – | + | D | 12 |
| 41 | 4 | F | – | – | + | D | 8 |
| 49 | 4 | M | nd | nd | – | D | 16 |
| 55 | 4 | F | + | + | + | D | 4 |
| 95 | 4 | F | + | + | + | D | 15 |
| 106 | 4 | F | + | + | + | N | 76+ |
| 107 | 4 | M | – | – | + | D | 10 |
| 112 | 4 | M | – | – | – | D | 18 |
| 114 | 4 | F | – | – | (+) | D | 25 |
| 123 | 4 | F | nd | + | + | D | 4 |
| 126 | 4 | M | – | + | + | D | 8 |
| 155 | 4 | F | + | – | – | D | 19 |
| 163 | 4 | M | + | + | + | D | 10 |
| 174 | 4 | F | + | + | + | D | 6 |
| St102 | 4 | M | + | + | + | D | 11 |
| St116 | 4 | F | – | + | – | A | 32+ |
| St118 | 4 | M | – | – | + | A | 24+ |
| St119 | 4 | M | + | – | (+) | A | 24+ |
| St126 | 4 | F | – | + | + | A | 25+ |
| St130 | 4 | F | (+) | + | + | D | 10 |
| St153 | 4 | M | nd | – | + | L | L |

Column 2: 1, 2A, 2B, 4S, 3 and 4, neuroblastoma stage; column 3: M, male; F, female; column 4: 1p-del, 1p deletion based on short tandem repeat polymorphism according to Martinsson et al, 1995; +, 1p deletion –, no 1p deletion (+), unsure results; nd, not determined; column 5: +, N-myc amplification; –, no amplification; nd, not determined; column 6: 17q gain, additional material of 17q, +, 17q gain >10% of counted cells; –, no 17q gain; (+), unsure results; column 7: N, no evidence of disease; D, dead of disease; A, alive with disease; DOC, dead of surgical complications; L, lost for follow-up; column 8: follow-up (months); +, alive at follow-up.

### MATERIALS AND METHODS

### Patients

Tumour samples were obtained from 48 children with neuroblastoma of all different stages (Table 1). The children were staged according to the International Neuroblastoma Staging System criteria (INSS, Brodeur et al, 1993). Tumour cell content of the samples was histologically assessed in adjacent tumour tissue to that used for DNA extraction. All tumour specimens were used for FISH analysis, and 43 from which DNA had been extracted were used for polymerase chain reaction (PCR)-based SSCP/HD detection. Of these, 32 were sequenced for the SSTR2 gene. In previous studies (Martinsson et al, 1995, 1997, and unpublished data), MYCN amplification and 1p deletion have been detected for most of the patients included in this study using FISH analysis and PCR-based DNA polymorphisms.

© 1999 Cancer Research Campaign

**Table 2** PCR primers used in this study

| Exon | Primer | Sequence | [a]Position 5′–3′ | Length of fragment (bp) |
|---|---|---|---|---|
| 1 | SSTR2-1FP | CTG GAA CTA GCC TAA GAC TGA AAA | 53–76 | 294 |
| | SSTR2-1RP | TGC GAT GGC CAG GTT GAG | 346–329 | |
| | SSTR2-2FP | GAA GAC CAT CAC CAA CAT TTA CAT | 304–337 | 333 |
| | SSTR2-2RP | CTC CGG AGC CCA GCA TA | 636–620 | |
| | SSTR2-3FP | ATG ATC ACC ATG GCT GTG T | 563–581 | 350 |
| | SSTR2-3RP | ACG TTG AAT ATG TAG AAG GGA AGC | 912–889 | |
| | SSTR2-4FP | ATC GTG GTG GCT GTC TTC | 860–877 | 345 |
| | SSTR2-4RP | CCC CCA AGC AGT TCA GA | 1204–1188 | |
| 2 | SSTR2-5FP | TTG AAT GAT AAT GTG CTA A | 7–25 | 276 |
| | SSTR2-5RP | CAT ACT CGA ATT TGC TAC T | 282–264 | |

[a] Exon 1, GenBank accession number M81830; alternatively spliced exon 2, GenBank accession number L13033.

### PCR-amplification

Forward (F) and reverse (R) primers for the SSTR2 gene (17q24) and marker D17S796 (17p) were synthesized using an ABI Applied Biosystem 392 DNA/RNA Synthesizer. SSTR2 exon 1, corresponding to the complete SSTR2 isoform 2a and approximately 94% of isoform 2b, and the alternatively spliced exon 2, corresponding to the remainder of SSTR2 isoform 2b (Patel et al, 1993), was amplified in five pieces using primers according to Table 2. Primer sequences for SSTR2 were selected using the DNASTAR primer select software (Lasergene, Madison, WI, USA) from the published cDNA sequences (GenBank accession numbers M81830 and L13033). Primers for D17S796 were from Dib et al (1996). Amplifications were carried out in 20-μl volume containing 50–100 ng template DNA; 15 pmol of each primer; 10 mM Tris–HCl (pH 8.3); 30 mM potassium chloride; 1.5 mM magnesium chloride; 0.001% (w/v) gelatin; 4.4 nmol of each dATP, dCTP, dGTP, dTTP, and 0.25 U *Taq* DNA polymerase (Pharmacia). Amplifications were performed with 30 standard cycles of 30–45 s at 94°C, 30–45 s at 58°C and 60 s at 72°C. Following the last cycle, an additional extension step was performed for 7 min at 72°C. SSTR2–4 was amplified in 30 μl volume containing 2.5 U *Taq* DNA polymerase with an initial incubation at 95°C for 5 min.

### Cytogenetic preparations

Touch prepared imprint slides from primary neuroblastoma tumours were subjected to standard procedures of hypotonic treatment (0.3% sodium chloride) for 10 min and fixation in increasing concentrations of ethanol:acetic acid (vol. 3:1) solution.

### Isolation of bacterial artificial chromosomes and labelling of probes for FISH

The Human bacterial artificial chromosomes (BAC) DNA pool library, purchased from Research Genetics Inc. (Huntsville, AL, USA), was screened with PCR-assays using primers for marker D17S796 (17p) and primers for the SSTR2 gene (17q24) respectively (Table 2). The BAC-DNA was extracted and purified using QIAGEN Plasmid Purification kit. The 17q-BAC (43–C7) was labelled with Cy3-dCTP and the 17p-BAC (146-G1) was labelled with FluoroX-dCTP with nick translation according to FluoroLink Cy3 Nick Translation Kit (Amersham Life Science). The labelled BAC-DNAs were used as FISH probes on touch prepared imprint slides.

### Fluorescence in situ hybridization

The probes used were Cy3 labelled BAC 43-C7 (17q24, giving a red signal), FluoroX labelled marker BAC 146-G1 (17p, giving a green signal), and digoxigenin/FITC-labelled p53 (17p13.1, giving a green signal) from Oncor. The probes were hybridized to metaphase chromosome control slides and to interphase slides from primary NB tumours. Slides were dehydrated in ethanol series, and denatured in sodium hydroxide (NaOH) solution (0.6% NaOH, 70% ethanol). The interphase slides were pre-washed in 70% acetic acid for 30 s, and dehydrated in both ethanol and acetone. BAC-probes were denatured for 10 min at 73°C in hybridization buffer (50% formamide, 10% dextran sulphate, 2 × SSC (standard saline citrate), final pH 7.0) and pre-hybridized in the presence of a 50-fold excess of Cot-1 DNA (Life Technologies) for 2 h at 37°C. The 17p-digoxygenin probe was pre-warmed at 37°C for 5 min. Ten microlitrss of each probe was applied to slides in mixtures and hybridization was performed under a 25 × 25 mm coverslip in a moist chamber at 37°C overnight.

After hybridization, slides were washed in 2 × SSC at 50°C. Slides hybridized with 17p-digoxygenin probe were immunochemically stained with fluorescein isothiocyanate (FITC)-conjugated anti-digoxygenin antibody (Oncor) under a coverslip in a moist chamber at 37°C for 20 min. DNA was stained in 0.25 μg $ml^{-1}$ 4,6-Diamidino-2-Phenylindole (DAPI, Sigma) in $Na_2HPO_4$-buffer, pH 7.0, and an anti-fade solution containing 2% 1,4 diazabicyclo-(2,2,2) octane (DABCO, Sigma), 69% glycerol, 2 mM Tris–HCl, pH 8.0, and 0.9 mg $ml^{-1}$ p-phenylenediamine (PPD, Sigma) was applied. The preparations were examined with a Zeiss Axiophot microscope with appropriate filters, photographed with a computer-driven IMAC-CCD colour camera and modified with the Meta system (Meta systems, Hard & Software GmbH, D68804 Altlussheim, Germany).

### SSCP/HD

The SSCP/HD electrophoreses were run on 20% homogeneous Phast gels with buffer system of native strips in Tris–tricin (0.2 M Tris, 0.2 M tricin) at 15°C, 400 V, 5.0 mA, 1.0 W for 250 AVh on a Phast system (Pharmacia Biotech). The Phast gels were pre-run

 © 1999 Cancer Research Campaign at 400 V, 5.0 mA, 1.0 W for 10–15 AVh. PCR-products were diluted two- to threefold with a formamide-dye solution containing 98% formamide, 0.05% xylene cyanol, 0.05% bromophenol blue, and 20 mM EDTA. The reaction mixtures were denatured for 5 min at 98°C, and approximately 1.0 µl of each sample was loaded on the gel. The Phast-gels were developed using Bio-Rad silver-stain (Bio-Rad Laboratories).

### DNA-sequencing

Sequencing was performed using either an ABI Prism 377 DNA Sequencer or an ABI Prism 310 Genetic Analyzer (Perkin-Elmer). The sequencing reactions were made using ABI Prism Big Dye Terminator Cycle Sequencing Ready Reaction Kit (Perkin-Elmer). The PCR-products were purified with QIAquick Spin PCR purification kit (QIAGEN), and the concentration of the PCR-products were estimated by comparison to a 100 bp-mass ladder on a 2% agarose gel. The sequence reactions were performed with 25 standard cycles of 45 s at 95°C, 45 s at 55°C and 180 s at 60°C using approximately 70 ng PCR-product and primers SSTR2-1FP, SSTR2-2FP, SSTR2-3RP, SSTR2-4RP, SSTR2-5RP respectively (Table 2). The sequence reaction products were precipitated with ethanol and the pellets were diluted in 'loading buffer' (5 vol. deionized formamide, 1 vol. 25 mM EDTA, pH 8.0, blue dextran) when using ABI Prism 377 DNA Sequencer or in 'Template Suppression reagent' TSr (Perkin-Elmer) when using ABI Prism 310 Genetic Analyzer. Before loading, the samples were denatured for 3 min at 95°C on a thermo block. The sequence reaction products, using ABI Prism 377 DNA Sequencer, were loaded on a 7% acrylamide gel.

### RESULTS

### Detection of 17q gain with FISH analysis

Tumour samples from patients with neuroblastoma ($n$ = 48, Table 1) were subjected to FISH analysis with probes for chromosome 17 (Figure 1). The 17p probes, BAC 146-G1 or p53 (Oncor), were used as references to the 17q probe (BAC 43-C7, 17q24), to distinguish 17q gain from trisomy, tetrasomy and polysomy of the complete chromosome 17. Forty to 100 cells were counted from each patient, except for case 114 and St119 where only 25 cells could be counted. All tumours were informative for all probes. 17q gain was defined as additional material in more than 10% counted cells, +. 17q gain was found in 31 out of 48 neuroblastoma tumours, whereas two of these (case 114 and St119) were defined as uncertain 17q gain patients, (+), due to the small amount of informative cells (Table 1). Most of the 17q gain tumours showed one additional signal of 17q, i.e. two 17p signals and three 17q signals (2+3), but some also showed two or more additional signals of 17q (2+4, 2+5) (Figure 1). Many of the tumours from patients with lower stages of neuroblastoma showed tetrasomy or polysomy for chromosome 17. Only six patients, five with 17q gain (3+4, 3+5) and one without (3+3), showed trisomy for chromosome 17. Five showed monosomy of 17p (1+3).

### Analysis of 17q gain and prognosis

17q gain was found more frequently in patients with stage 4 (17 out of 21) compared with other stages combined (14 out of 26, $P$ = 0.028 Fisher's exact test, Table 1). Only one of the patients with stage 1 showed 17q gain. Five out of seven of the patients with stage 2 (stage 2A and 2B) had 17q gain, while seven out of the 11 patients with stage 3 had additional material of 17q. Forty-five out of 48 children were evaluated for survival probability in relation to 17q gain. Two children were lost for follow-up and one died of surgical complications, and were therefore not included. Forty children had tumours informative for all three prognostic factors, i.e. 1p-deletion, MYCN amplification and 17q gain, and these results are displayed in the Venn diagrams (Figure 2). Children with tumours of all different stages showing 17q gain had a worse outcome, 18 of 29 dead of disease versus three of 16 among those with tumours with no 17q gain. Survival probability according to Kaplan–Meier was also better for those with no 17q gain, 78.6% at 5 years from diagnosis compared to 34.3% for those with 17q gain ($P$ = 0.0009, Mantel–Haentzel log-rank test, Figure 2A). Children with tumours at favourable stage (stage 1, 2, 3 and 4S) showing 17q gain had a significantly worse outcome, six of 13 dead of disease versus zero of 12 among those with tumours showing no 17q gain. Thus, the survival probability of low-stage tumours showing no 17q gain was 100% at 5 years from diagnosis compared to 52.5% for those showing 17q gain ($P$ = 0.0021, Mantel–Haentzel log-rank test, Figure 2B). As displayed in the Venn-diagram no low-stage tumour showed 1p-deletion or MYCN amplification without concomitant 17q gain (Figure 2B). Children with high-stage tumours (stage 4) showing 17q gain had no significantly worse outcome, 12 of 16 dead of disease versus three of four among those with tumours showing no 17q additional material (Figure 2C).

### Mutation detection of the SSTR2 gene with PCR-based SSCP/HD and sequencing

Tumour samples from patients with neuroblastoma ($n$ = 43), were subjected to PCR-based SSCP/HD analysis for SSTR2 exon 1 (data not shown). Twenty-two of the 43 neuroblastomas were stage 4 tumours, ten were stage 3 tumours and nine were of unknown stages. The SSTR2 exon 1 was amplified from tumour DNA and normal DNA in four overlapping parts, giving approximately 300–350 bp long fragments (Figure 3). SSCP patterns from tumour DNA of patients were compared with SSCP patterns from the patients constitutional DNA (prepared from blood lymphocytes) and that of control DNA subsequently sequenced.

Generally, most of the SSCP pattern generated from the same SSTR2 fragment were identical, and did not diverge notably from normal controls. Sequencing was carried out for the complete SSTR2 cDNA sequence (i.e. exon 1 and alternatively spliced exon 2, Figure 3) for 18 PCR-samples, while an additional 14 tumour samples were partially sequenced (1–4 fragments of the total 5 fragments). All sequenced were compared to the SSTR2 sequence derived from the genome database (GenBank accession numbers M81830 and L13033), and none of them showed any deviation from the reference sequence.

### DISCUSSION

Neuroblastoma is an extremely heterogeneous disease, and a number of genetic and cytogenetic alterations have shown to associate with prognosis. In the present study, FISH was used to study status of chromosome 17q in 48 neuroblastoma tumours. 17q gain

© 1999 Cancer Research Campaign 

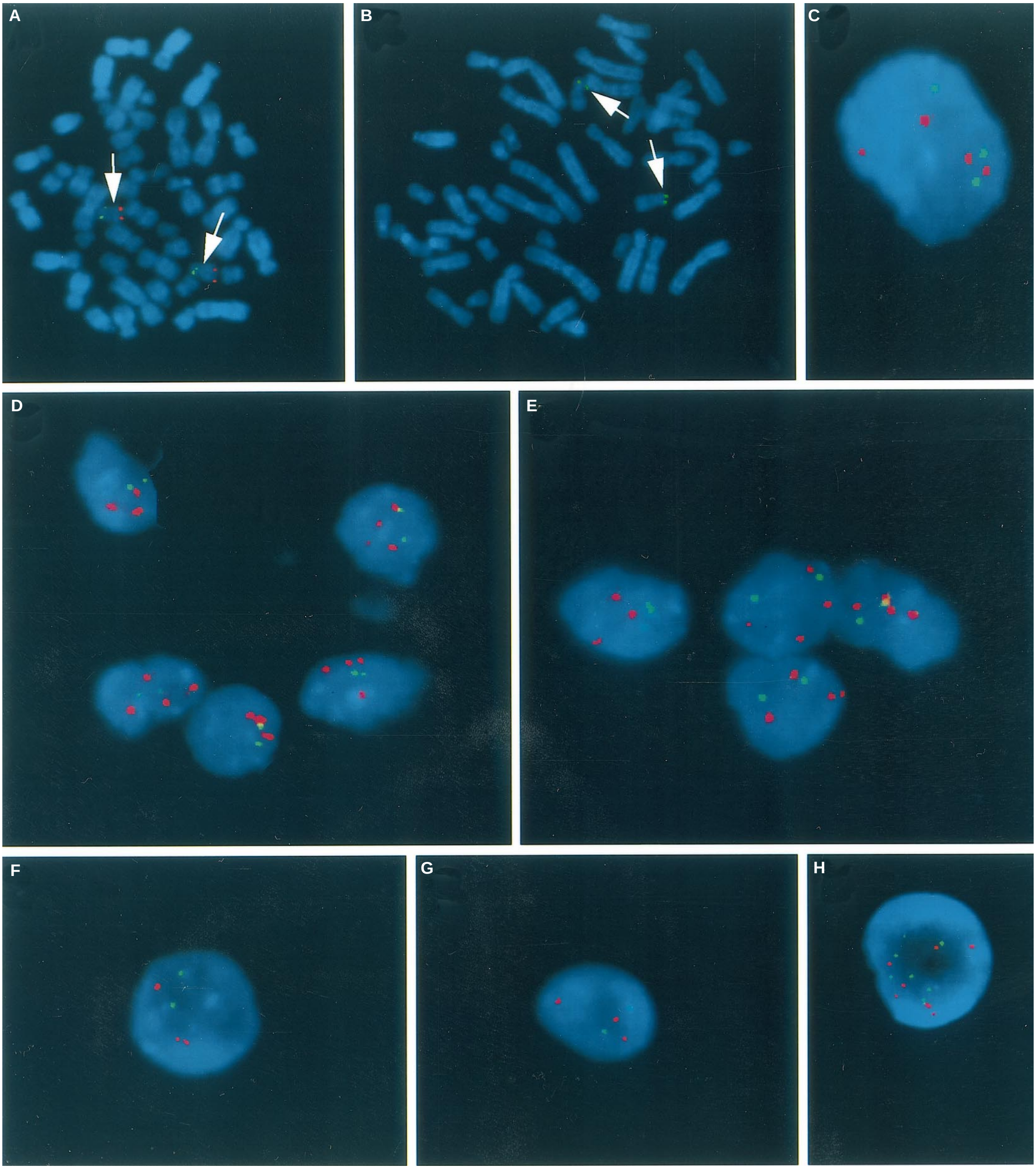

**Figure 1** Representative FISH analyses of normal cells (**A–B**) and neuroblastoma tumour cells (**C–H**). (**A**) Metaphase chromosomes 17q 24 (BAC 43–C7): red/17p13.1 (p53): green. (**B**) Metaphase chromosomes 17p (BAC 146-G1): green. (**C**) 17q gain (3+4) interphase cell, case 138, 17q: red/17p:green. (**D**) 17q gain (2+3, 2+4) interphase cells, case 95, 17q: red/17p: green. (**E**) 17q gain (2+3, 2+4) interphase cells, case 121, 17q: red/17p: green. (**F**) 17q gain (2+3) interphase cell, case St118, 17q: red/17p:green. (**G**) 17q gain (2+3) interphase cell, case 136, 17q: red/17p: green. (**H**) Interphase cell showing polysomy for chromosome 17, case 84, 17q: red/17p: green. The digital images were captured and modified using Meta Systems (Meta Systems, Hard & Software GmbH, D68804 Altlussheim, Germany)

was detected in 65% of the neuroblastoma tumours. Moreover, 17q gain was found to be associated with poor prognostic factors such as deletion of chromosome arm 1p and amplification of the MYCN oncogene. These findings are supported by earlier studies (Lastowska et al, 1997*a*; Plantaz et al, 1997). The association between 1p deletion and 17q gain is not surprising since physical

 *© 1999 Cancer Research Campaign*

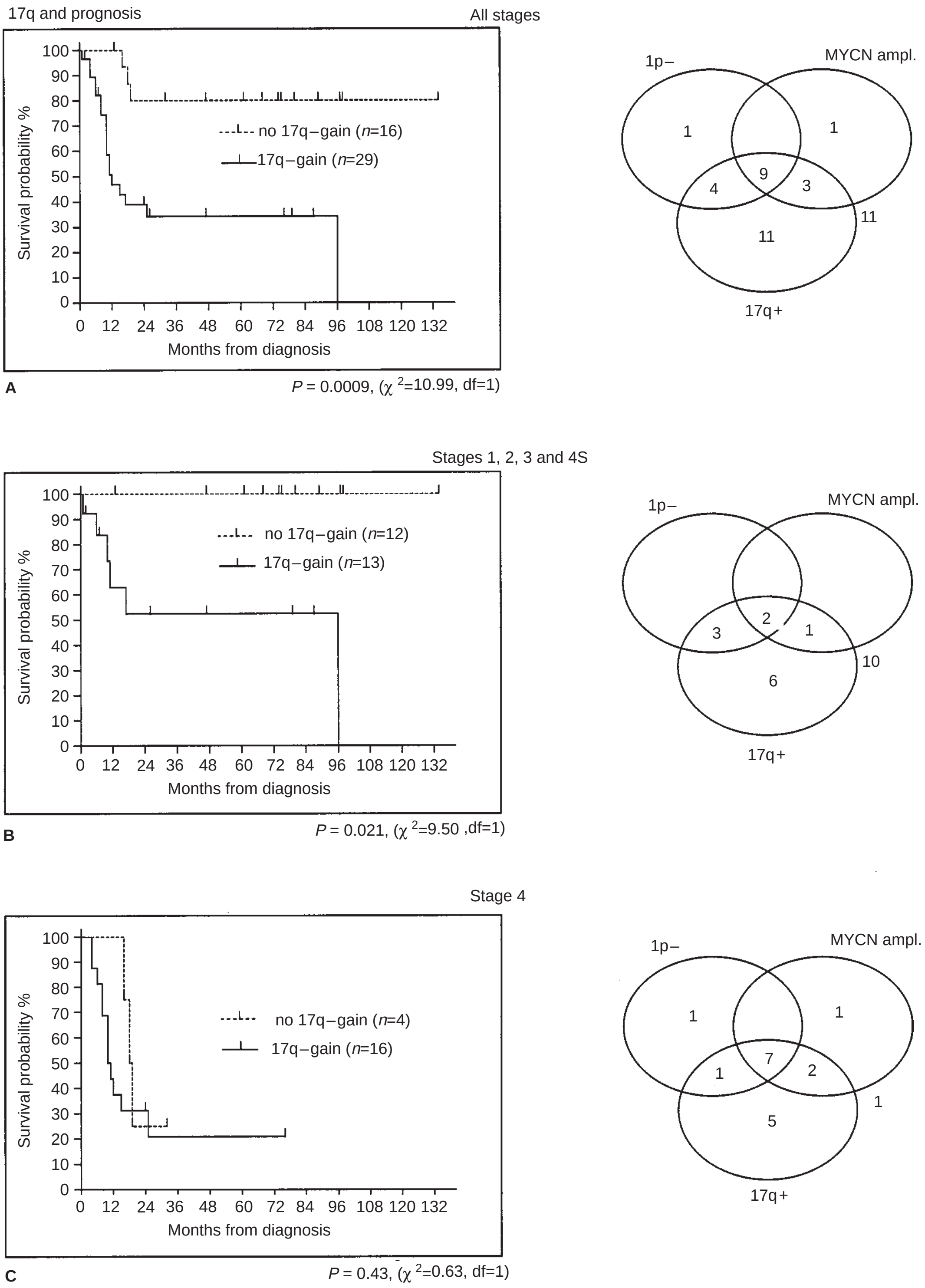

**Figure 2** Survival probability according to Kaplan–Meier (left) combined with Venn diagrams (right) of the relationship between the prognostic factors 1p-deletion, MYCN amplification, and 17q gain in neuroblastoma patients. (**A**) Survival probability for children with all stages of neuroblastoma; with 17q gain (solid line) and without 17q-gain (dashed line). (**B**) Survival probability for children with tumours of favourable stages 1, 2, 3, and 4S. (**C**) Survival probability for children with neuroblastoma stage 4 tumours. Only patients informative for all prognostic factors were used in the Venn diagrams connection between these chromosomes has been seen in the form of unbalanced translocations der(1)t(1p;17q) (Caron et al, 1994; Van Roy et al, 1994; Meddeb et al, 1996; Lastowska et al, 1997*a*, 1997*b*). These translocations result in loss of 1p and gain of 17q, and are thought to occur in the S/G2 phase of the cell cycle (Caron et al, 1994). In the present study, FISH was only carried out on interphase cells, and therefore the frequency of such translocation could not be investigated. The association between MYCN

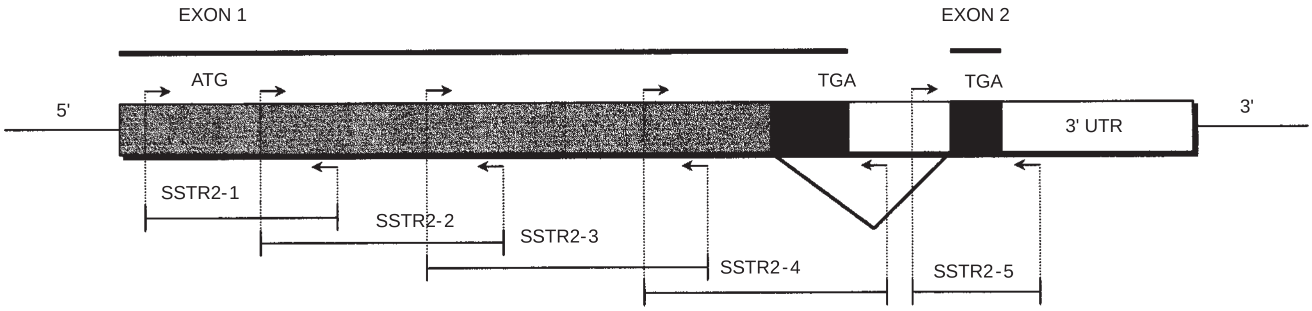

**Figure 3** Organization of the SSTR2 gene according to Patel et al (1993), showing the two spliced variants. The shaded box represents the unspliced transcript. The black boxes represent the coding sequences of the variable C-terminal regions of the two transcripts. The white areas represent transcribed but untranslated DNA. The predicted translational start (ATG) and stop (TGA) codons are illustrated. The arrows represent the primers; forward primers in 3′ direction and the reverse primers in 5′ direction. SSTR2, 1–5 represents the five amplified fragments amplification and 17q gain is more difficult to explain. There are some few cases where MYCN HSRs are flanked by 17q material (Van Roy et al, 1994; Lastowska et al, 1997*a*). But such rearrangements are not generally seen in primary tumours, where MYCN amplifies mainly in the form of double minute chromosomes (Lastowska et al, 1997*a*). As found in the present study and in earlier reports, all these three alterations; 1p-deletion, MYCN amplification, and 17q gain, show strong association to each other. This gives rise to speculations whether they are structurally related in some kind of way. Like two earlier studies (Meddeb et al, 1996; Lastowska et al, 1997*a*) we also found that the number of 17q gain (31 out of 48 analysed neuroblastomas) exceeded the number of 1p deletions (14/40) and the number of MYCN amplifications (16/46). But not all of the 1p deletions and MYCN amplifications status were determined in this study, which has to be taken into account. Nevertheless, these results indicate that 1p deletion and MYCN amplification could be secondary events of 17q gain as a primary event.

In the present study most of the 17q gain tumours showed 1 additional signal of 17q (2+3), but 36% of the 17q gain tumours also showed two or more additional signals (2+4, 2+5) of 17q (Figure 1). This gave rise to questions about 17q gain being a dose-dependent feature. However, in this study no differences in survival probability between patients showing one additional 17q signal in relation to patients showing two or more additional 17q signals in the tumour specimens could be seen.

In the present study, five patients who died of disease had 17q gain without 1p deletion and MYCN amplification (cases 69, 32, 41, 107 and 114; Table 1), but there were also a number of patients surviving with no evidence of disease that showed 17q gain (e.g. cases 181, St156, 127, 138 and 106). However, from the data it was clear that children with tumours of all different stages showing 17q gain had a significantly worse outcome, 18 of 29 dead of disease versus three of 16 among those with tumours with no 17q additional material. Survival probability according to Kaplan–Meier was also better for those with no 17q gain, 75% at 5 years from diagnosis compared to 34% for those with 17q gain ($P$ = 0.0009, Figure 2A). Remarkably, among children with low-stage tumours (stage 1, 2, 3 and 4S) only those showing 17q gain died of disease (six of six), whereas none of those showing no 17q gain died from disease ($P$ = 0.0021, Figure 2B). In contrast, children with high-stage tumours (stage 4) showing 17q gain had no significantly worse outcome, 12 of 16 dead of disease versus three of four among those with tumours showing no 17q additional material. These results suggest that 17q gain as a prognostic factor plays a more crucial role in low-stage tumours, and that metastic stage 4 tumours display many other adverse prognostic factors (1p-deletions, MYCN amplifications, etc.) which somehow surpass the effect of 17q gain. As seen in the Venn-diagram no low-stage tumour and only two of the high-stage tumours showed 1p-deletion or MYCN amplification without showing 17q gain as well (Figure 2B). This supports the hypothesis of 17q gain as a primary genetic event.

Two studies, one by in vivo detection with $^{111}$In-pentetreotide scintigraphy (Schilling et al, 1998) and one by in vitro detection with competitive reverse transcription PCR (RT-PCR) (Sestini et al., 1996), have shown that expression of SSTR2 in neuroblastoma tumours is positively related to survival and negatively related to poor prognosis. A point mutation in the SSTR2 gene, causing loss of 182 C-terminal amino acid residues of SSTR2, has earlier been detected in a human small-cell lung cancer carcinoma (Zhang et al, 1995). In the present study we wanted to investigate whether the low level of SSTR2 expression in unfavourable neuroblastomas is a primary genetic event as described for the point mutation in the SSTR2 gene in small-cell lung cancer. Screening of 43 neuroblastoma tumours for mutations in the SSTR2 gene was carried out with PCR-based SSCP/HD and sequencing. We found that all SSCP patterns were normal compared to the wild-types, and no mutations could be detected in the SSTR2 gene of the 32 successfully sequenced patients as a contrast to the reported SSTR2 gene mutation earlier detected in a colon cancer cell line (Zhang et al, 1995). This suggests that mutations in the SSTR2 gene is uncommon in neuroblastoma tumours and not related to the 17 gain often seen in these.

In conclusion, the neuroblastoma patients analysed in this study have a high incidence of 17q gain in agreement with what have been reported by other groups. 17q gain is associated with poor prognosis and with other prognostic factors. No mutations could be detected in the SSTR2 gene that explains its reported low expression in high-stage neuroblastoma.

### ACKNOWLEDGEMENTS

We gratefully acknowledge the financial support of the Swedish Cancer Society, the Children’s Cancer foundation and the King Gustav V Jubilee Clinic Cancer Research foundation.

 © 1999 Cancer Research Campaign

### REFERENCES

Ambros PF, Ambros IM, Strehl S, Bauer S, Luegmayr A, Kavar H, Ladenstein R, Fink FM, Horcher E, Printz G, et al (1995) Regression and progression in neuroblastoma. Does genetics predict tumour behaviour? *Eur J Cancer* **31A**: 510–515

Bell GI, Yasuda K, Kong H, Law SF, Raynor K and Reisine T (1995) Molecular biology of somatostatin receptors. *Ciba Found Symp* **190**: 65–88

Brodeur GM (1998) Clinical and biological aspects of neuroblastoma. In: *The Genetic Basis of Human Cancer*, Vogelstein B and Kinzler KW (eds), pp. 691–711. McGraw-Hill: New York

Brodeur GM, Pritchard J, Berthold F, Carlsen NL, Castel V, Castelberry RP, De Bernardi B, Evans AE, Favrot M, Hedborg F, et al (1993) Revisions of the international criteria for neuroblastoma diagnosis, staging, and response to treatment. *J Clin Oncol* **11**: 1466–1477

Caron H, van Sluis P, van Roy N, de Kraker J, Speleman F, Voute PA, Westerveld A, Slater R and Veersteg R (1994) Recurrent 1;17 translocations in human neuroblastoma reveal nonhomologous mitotic recombination during the S/G2 phase as a novel mechanism for loss of heterozygosity. *Am J Hum Genet* **55**: 341–347

Caron H, van Sluis P, van Roy N, de Kraker J, Bokkerink J, Egeler M, Laureys G, Slater R, Westerveld A, Voute PA and Versteeg R (1996) Allelic loss of chromosome 1p as a predictor of unfavourable outcome in patients with neuroblastoma. *N Engl J Med* **334**: 225–230

Dib C, Faure S, Fizames C, Samson D, Drouot N, Vignal A, Millasseau P, Marc S, Hazan J, Seboun E, Lathrop M, Gyapay G, Morissette J and Weissenbach J (1996) A comprehensive genetic map of the human genome based on 5264 microsatellites. *Nature* **380**: 152–154

Gale G, D'Angio G, Uri A, Chatten J and Koop CE (1982) Cancer in neonates: the experience at the children's hospital of Philadelphia. *Pediatrics* **70**: 409–413

Kogner P, Borgström P, Bjellerup P, Schilling FH, Refai E, Jonsson C, Dominici C, Wassberg E, Bihl H, Jacobsson H, Theodorsson E and Hassan M (1997) Somatostatin in neuroblastoma and ganglioneuroma. *Eur J Cancer* **33**: 2084–2089

Lastowska M, Cotterill S, Pearson ADJ, Roberts P, McGuckin A, Lewis I and Bown N (1997*a*) Gain of chromosome arm 17q predicts unfavorable outcome in neuroblastoma patients. *Eur J Cancer* **33**: 1627–1633

Lastowska M, Roberts P, Pearson ADJ, Lewis I, Wolstenholme J and Bown N (1997*b*) Promiscuous translocations of chromosome arm 17q in human neuroblastomas. *Genes Chromosomes Cancer* **19**: 143–149

Lastowska M, Van Roy N, Bown N, Speleman F, Lunec J, Strachan T, Pearson ADJ and Jackson MS (1998) Molecular cytogenetic delineation of 17q translocation breakpoints in neuroblastoma cell lines. *Genes Chromosomes Cancer* **23**: 116–122

Look AT, Hayes FA, Shuster JJ, Douglass EC, Castleberry RP, Bowman LC, Smith EI and Brodeur GM (1991) *J Clin Oncol* **9**: 581–591

Martinsson T, Sjöberg RM, Hedborg F and Kogner P (1995) Deletion of 1p loci and microsatellite instability in neuroblastomas analyzed with short-tandem repeat polymorphism. *Cancer Res* **55**: 5681–5686

Martinsson T, Sjöberg RM, Hallstensson K, Nordling M, Hedborg F and Kogner P (1997) Delimitation of a critical tumor suppressor region at distal 1p in neuroblastoma tumors. *Eur J Cancer* **33**: 1997–2001

Meddeb M, Danglot G, Chudoba I, Vénaut AM, Bénard J, Avet-Loiseau H, Vasseur B, Le Paslier D, Terrier-Lacombe MJ, Hartmann O and Bernheim A (1996) Additional copies of a 25 Mb chromosomal region originating from 17q23.1–17qter are present in 90% of high-grade neuroblastomas. *Genes Chromosomes Cancer* **17**: 156–165

Patel YC, Greenwood MT, Kent G, Panetta R and Srikant CB (1993) Multiple gene transcripts of the somatostatin receptor SSTR2: Tissue selective distribution and cAMP regulation. *Biochem Biophys Res Commun* **192**: 288–294

Patel YC, Greenwood MT, Panetta R, Demchyshyn L, Niznik H and Srikant CB (1995) The somatostatin receptor family. *Life Sci* **57**: 1249–1265

Plantaz D, Mohapatra G, Matthay KK, Pellarin M, Seeger RC and Feuerenstein BG (1997) Gain of chromosome 17 is the most frequent abnormality detected in neuroblastoma by comparative genomic hybridization. *Am J Pathol* **150**: 81–89

Schilling FH, Ambros PF, Bihl H, Martinsson T, Ambros IM, Borgström P, Jacobsson H, Falkmer UG, Treuner J and Kogner P (1998) Somatostatin receptor expression in vivo is absent in neuroblastomas showing distal deletion of chromosome 1p and di/tetraploid DNA content. *Eur J Cancer* (in press)

Seeger RC, Brodeur GM, Sather H, Dalton A, Siegel SE, Wong KY and Hammond D (1985) Association of multiple copies of the N-myc oncogene with rapid progression of neuroblastomas. *N Eng J Med* **313**: 1111–1116

Sestini R, Orlando C, Peri A, Tricarico C, Pazzagli M, Serio M, Pagani A, Bussolati G, Ganchi S and Maggi M (1996) Quantitation of somatostatin receptor type 2 gene expression in neuroblastoma cell lines and primary tumors using competitive reverse transcription-polymerase chain reaction. *Clin Cancer Res* **2**: 1757–1765

Vandesompele J, Van Roy N, Van Gele M, Laureys G, Ambros P, Heimann P, Devalck C, Schuuring E, Brock P, Otten J, Gyselinck J, De Paepe A and Speleman F (1998) Genetic heterogeneity of neuroblastoma studied by comparative genomic hybridization. *Genes Chromosomes Cancer* **23**: 141–152

Van Roy N, Laureys G, Cheng NG, Willem P, Opdenakker G, Versteeg R and Speleman F (1994) 1;17 translocations and other chromosome 17 rearrangements in human primary tumor neuroblastoma tumors and cell lines. *Genes Chromosomes Cancer* **10**: 103–114

Yamada Y, Post SR, Wang K, Tager HS, Bell GI and Seino S (1992) Cloning and functional characterization of a family of human and mouse somatostatin receptors expressed in brain, gastronal tract, and kidneys. *Proc Natl Acad Sci USA* **89**: 251–255

Zhang CY, Yokogoshi Y, Yoshimoto K, Fujinaka Y, Matsumoto K and Saito S (1995) Point mutation of the somatostatin receptor 2 gene in the human small cell lung cancer cell line COR-L103. *Biochem Biophys Res Commun* **210**: 805–815

© 1999 Cancer Research Campaign
